# Sanguinarine

**Published:** 1910-02

**Authors:** George L. Servoss

**Affiliations:** Fairview, Nevada


For Texas Medical Journal.

### Sanguinarine.

BY GEORGE L. SERVOSS, M. D., FAIRVIEW, NEVADA.

In a recent issue of this journal emetine, one of the active principles of ipecac, was considered. Sanguinarine, the alkaloid from bloodroot, or *Sanguinaria Canadensis,* is employed in many cases in which emetine is applied, especially in pulmonary affections where emetine fails to excite a sufficient expectorant effect. In medicine sanguinarine is employed as the nitrate salt, and in addition to acting as a stimulant expectorant, increases the digestion, strengthens the heart and slows the pulse, when administered in small doses, while large doses are followed by nausea, sedation of the heart with slowing of the pulse, increase of the perspiration, expectoration, urine and bile. Sanguinarine in large doses also increases the menstrual flow. Very large doses are followed by violent vomiting and at times catharsis, with vertigo, troubled vision and great prostration, with increased salivary flow. This is followed by extreme gastro-intestinal irritation and symptoms of collapse, depression of the spinal reflexes and death from paralysis of the respiratory and cardiac centers.

The eclectics employ sanguinarine in conjunction with lobelia as an emetic in febrile conditions to clear the stomach and arouse the liver and other glandular activity. It is employed in chronic hepatitis, atonic dyspepsia, gastric and duodenal catarrh and jaundice, sick headache and torpid liver, rheumatism, dysentery and scrofula with faulty circulation; amenorrhea, associated with anemia and dysmenorrhea with debility, and has been found available in the treatment of syphilides, tinea and hypertrophic rhinitis (Felter and Lloyd).

The most prominent use of sanguinarine is as a stimulant expectorant. It arouses the sensibilities and makes the patient cough, thereby ejecting the secretions and relieving the overloaded mucous membranes of the respiratory tract. It is indicated in the low conditions frequently encountered in pneumonia, bronchitis, phthisis and capillary bronchitis, when the sensibility of the respiratory mucosa is low and the patient makes no effort to get relief by coughing. In such cases the mucosa is stimulated and by coughing harder the patient throws off the accumulation of mucus and does not "drown in his own sputum." Many cases have been carried through to a happy termination by the employment of sanguinarine promptly when the conditions have indicated, and it has become a very valuable remedy in the diseases above mentioned. Care should be taken not to exceed the stimulant dose, as the sedation and nausea of large or overdoses are not desirable in such cases. Employed judiciously, sanguinarine will be found a most reliable remedy and one which will be employed with greater frequency as the practician realizes its possibilities and effects.

Sanguinarine has been found valuable in acute respiratory catarrhs with low vitality of the tissues. In such cases it excites the tissue vitality. It has been found useful in acute pharyngitis, acute and chronic nasal catarrhs, humid asthma, whooping-cough, membranous croup and the sore throat of scarlatina.

The authorities give the specific indications for sanguinarine: Tickling or irritation of the throat, with cough, burning or irritation in fauces, larynx, pharynx or nose, with red surface and thin, acrid, burning or smarting discharges, dryness of nasopharynx or throat and sharp lancinating pain, and a feeling as if the walls of the throat rubbed against each other; poststernal constriction, uneasiness and burning in stomach with nervousness.

Too much stress can not be placed upon the application of sanguinarine in the low conditions in pneumonia and bronchitis, where the patient is in an apathetic condition, making no effort to obtain relief by coughing to rid himself of the accumulated secretions. When the drug is employed in physiological dosage coughing follows and the relief is marked, death by suffocation being frequently averted.

As a stimulant expectorant the dose of sanguinarine nitrate is 1-67 to 1-22 grain every hour to effect relief or slight nausea. In anemic amenorrhea, 1-22 to 1-12 grain before meals and at bedtime, during the intermenstrual period, with iron arsenate, adding apiol during the menstrual week.

